# Ocular biological parameters and prevalence of myopia in vocational high school and general high school in China

**DOI:** 10.3389/fpubh.2023.1100437

**Published:** 2023-03-20

**Authors:** Yang Liu, Dexin Meng, Yun Wang, Xuechun Wang, Caihong Xue, Rui Hao, Wei Zhang

**Affiliations:** ^1^Tianjin Eye Hospital, Tianjin Key Lab of Ophthalmology and Visual Science, Nankai University Affiliated Eye Hospital, Clinical College of Ophthalmology Tianjin Medical University, Tianjin, China; ^2^Department of Ophthalmology, The Second Affiliated Hospital of Harbin Medical University, Harbin, China; ^3^Tianjin Occupational Diseases Precaution and Therapeutic Hospital (Tianjin Workers’ Hospital), Tianjin, China

**Keywords:** prevalence, ocular biometry, myopia, high school students, educational schedule

## Abstract

**Significance:**

Higher prevalence of myopia is possibly associated with more extended schooling schedules. Therefore, adjustments to high school curricula may aid in reducing the prevalence of myopia among adolescents.

**Purpose:**

To investigate the prevalence of myopia among 15- to 18-year-old adolescents in Tianjin, China, and to evaluate the impact of different educational schedules on the prevalence of myopia among high school students.

**Methods:**

This is a school-based epidemiological study with a cross-sectional design. Ocular biological parameters and noncycloplegic photorefraction were examined using optical biometry devices and photoscreener devices. Each student’s spherical equivalent (SE) and ocular biometry were recorded, and the prevalence of myopia was calculated.

**Results:**

A total of 2,867 participants (1,519 males and 1,348 females) were tested for non-cycloplegic refraction, axial length (AL), central corneal thickness (CCT), anterior chamber depth (ACD) and lens thickness (LT). In this research, the overall prevalence of myopia was 81.6%, with high myopia accounting for 11.8%. Myopia prevalence was substantially higher in general high schools than in vocational high schools, with 86.1 and 70.1%, respectively. There were no significant differences in the prevalence of myopia (*p* = 0.744) or high myopia (*p* = 0.851) across the three vocational school years. In the general high school, however, there was an increase of 4.6% (*p* < 0.05) in myopia prevalence between year 10 and year12.

**Conclusion:**

Comparing vocational and standard high school students, there are considerable disparities in prevalence of myopia, spherical equivalent, and ocular biological parameters. The prevalence of myopia and high myopia increased among standard high school students, but remained relatively consistent among students in vocational schools.

## Introduction

Myopia is a multifactorial condition that has substantial medical and economic consequences for those affected, as well as for society as a whole. Because of the ocular comorbidities associated with severe myopia, such as rhegmatogenous retinal detachment, myopic macular degeneration, premature cataract, and glaucoma, severe myopia is a leading cause of visual impairment globally (1). In 2050, it is anticipated that 4,758 million individuals will have myopia, and 938 million will have severe myopia, representing approximately 50 and 10% of the global population, respectively (2). Both inherited and environmental variables have been implicated in the etiology of myopia. Near-work and outdoor activities throughout childhood, education, and residency (urban vs. rural) have all been related to the varied incidence and severity of myopia (3). Near work has long been thought to be a potential mediator for the connection between high myopia prevalence and increased educational intensity due to high accommodating demand (4). However, the precise reasons and mechanisms remain unclear. Prior studies on high school education have been limited to general high school students, with no vocational high school students of the same age included (5, 6). According to data issued by the National Bureau of Statistics of China in 2018, vocational education is a significant destination for junior high school graduates, with 15.925 million students, or 40.10% of high school students enrolled (7). In addition, the target group for myopia prevention and control is typically kindergarten through elementary school pupils. But adolescents aged 15 to 18 who attend high school are still in a crucial stage of myopia development. To contribute more effectively to the control and prevention of myopia in teenagers, it is required to research the prevalence of myopia and ocular biological parameters among high school students, as well as the association between myopia and educational patterns.

## Materials and methods

### Study settings and participants

We conducted a cross-sectional study based on a multistage stratified cluster random sampling in a suburban district of Tianjin, from October to December 2019. A total of 2,956 children from 5 schools (2 vocational high schools and 3 general high schools) participated in the survey. This school-based cross-sectional study was approved by the ethics board of Tianjin Eye Hospital, Tianjin, China. Written informed consent was obtained before the start of the study from the parents of all participants.

Prior to data collection, participants were screened to determine their eligibility for the experiment. Children with a history of ocular or systemic disease, current use of systemic or ocular medications that may modify refractive status, history of previous ophthalmic surgery, or substantial binocular vision, accommodative abnormalities, or ocular movement disorders were excluded from the study. At least 1 month in advance, schools were informed of the screening date. For screening, all individuals were instructed to remove their prescription refractive spectacles. Students who regularly wore rigid contact lenses or received corneal refractive therapy (Ortho-K) were instructed to cease using them 4 weeks prior to the day of the examination. Students who wore soft contact lenses were urged to stop wearing them 3 days before the assessment.

### Chinese high schools and training system

**Figure 1:**
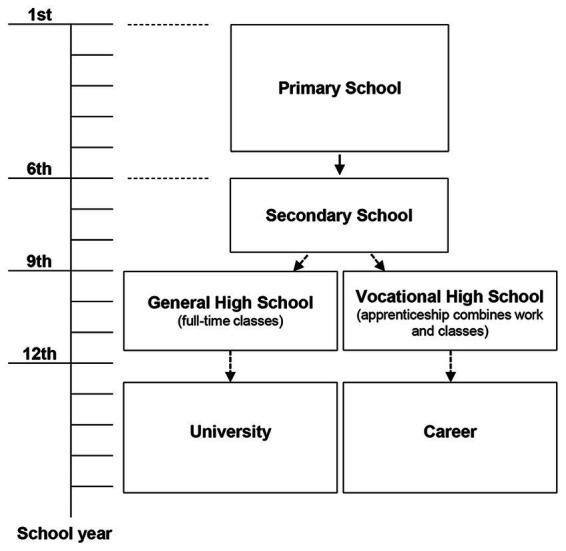
Demonstration of Chinese school system prior university.

The Chinese educational system is comprised of 6 years of elementary school, 3 years of both lower and upper secondary high school, and 4 years of the standard university curriculum (Figure 1). After completing lower secondary school, adolescents can select between two educational tracks (general high school or vocational high school) based on their scores on high school entrance exams. Students with better academic performance have a greater chance of being admitted to general high schools. Typically, students in general high schools continue their academically-focused education to obtain a regular high school diploma, allowing them to apply to traditional universities. In vocational high schools, however, major efforts are undertaken to provide technical education to students in order to obtain employment following graduation. Due to the difference in teaching purposes, the curriculum of vocational high schools will place less emphasis on academic learning and more attention on skill development.

### Refraction and ocular examinations

The ophthalmological examinations began at 09:00 a.m. for all participants to minimize daily fluctuations of the ocular biometrics by the biorhythm. All of the data was collected in a non-cycloplegic state by well-trained examiners. An optical biometry (SW9000, SUOER, China) was used to measure axial length, central corneal thickness, anterior chamber depth and lens thickness. The noncycloplegic refractive error was tested using the SW800 vision screener (SUOER, China), which is a recently developed photoscreener designed specifically for the Chinese population. The measurements of Spot and SW800 photoscreeners showed a strong agreement with cycloplegic retinoscopy refraction tests (8). The testing was undertaken by trained personnel, all measurements were repeated three times, and the average value of each parameter was calculated. In brief, the examiner held the photoscreener at a distance of 1 M from the child in a dark environment. The spherical equivalent refraction of the child was recorded automatically for both eyes. The screener’s measuring range was restricted to −7.5–+7.5 diopters (D) in 0.25 D increments. The software algorithm of the photoscreener would flag a referral for a complete eye examination and record data if significant refractive error (the refraction was out of range), anisometropia, or strabismus were detected. And then, the non-cycloplegic retinoscopy (REF 18240, Welch Allyn, United States) was performed by a senior optometrist. Spherical equivalent (sphere power + cylinder power/2) was used to calculate refraction. When spherical equivalent (SE) ≤ −0.50D, a subject was classified as myopic; myopic patients were further classified as mild (−3.00D < SE ≤ −0.50D), moderate (−6.00D < SE ≤ −3.00 D), and high (SE ≤ −6.00 D) myopic (9). Anterior chamber depth was defined as the distance between the corneal endothelium and the lens. The axial length was defined as the distance from the anterior corneal pole to an interference peak corresponding to the posterior retinal pole. Lens position is defined as the sum of the aqueous depth and half of the lens thickness.

### Statistical analysis

As the correlation coefficients of spherical equivalent (*R* = 0.92, *p* < 0.001) and axial length (*R* = 0.95, *p* < 0.001) were high in both left and right eyes, only data from the right eyes was used in the analysis. For the analysis, the statistical package R software for Windows and SPSS version 25.0 software (SPSS Inc., Chicago, IL, United States) were used for the statistical package. A normality test (Kolmogorov–Smirnov test) was performed. Logistic regression was carried out to investigate the effect of years, gender and two educational systems on the prevalence of myopia. A two-sided *p* < 0.05 was considered statistically significant at the 95% confidence interval (CI, a range of values defined such that there is a specified probability that the value of a parameter occurs inside it) level. The odds ratio (OR) quantifies the relationship between an exposure and an outcome.

## Results

A total of 2,867 students were enrolled in this research, with a mean age of 16.33 ± 1.0 (range 14–19 years). Among the subjects, 814 were from vocational high schools (mean age 16.48 ± 1.06, range 14–19 years) and 2053 (mean age 16.27 ± 0.97, range 14–19 years) from general high schools. The proportion of males was 53% (1,519 subjects). Among the subjects, 5 (0.6%) of vocational high school students and 27 (1.3%) of general high school students were out of the measurement range. Eighty-nine students were eliminated from the study regarding systemic or visual abnormalities such as amblyopia, strabismus, or other disorders that might alter the results.

Sample size, age, sex distribution, SE, prevalence rate of myopia and high myopia are shown in Table 1. In this school-based screening, the prevalence of myopia was 81.6% overall, and the prevalence of high myopia was 11.8%. In the general high schools, the prevalence of myopia was higher than that in the vocational high schools, at 86.1% versus 70.1%, respectively. Similarly, the prevalence of high myopia was higher in general high schools (13.7%) than in vocational schools (6.8%). Both disparities between the two schools were statistically significant (*p* < 0.001). In addition, the SE in general high schools was lower than in vocational high schools (*p* < 0.001): −2.50 D (IQR = 3.25), compared −3.88 D (IQR = 3.38). In all three school years, there were substantial differences in the SE and the prevalence of myopia of the two schools (*p* < 0.05). After adjusting for differences in age and gender using logistic regression, the overall prevalence of myopia among high school pupils remained elevated (OR = 2.55, 95% CI: 2.09–3.11; *p* < 0.001), and the year 10 (OR = 2.17, 95% CI: 1.54–3.05; *p* < 0.001), year 11 (OR = 2.03, 95% CI: 1.40–2.96; *p* < 0.001), and year 12 (OR = 3.39, 95% CI: 2.42–4.73; *p* < 0.001) were higher, respectively (Table 2). The overall amount of high myopic students was likewise greater (OR = 2.19, 95% CI: 1.61–3.00; *p* < 0.001), and the year 10 (OR = 1.65, 95% CI: 1.02–2.77; *p* = 0.048), the year 11 (OR = 1.93, 95% CI: 1.08–3.43; *p* = 0.026) and year 12 (OR = 2.53, 95% CI: 1.59–4.02; *p* < 0.001) were higher, respectively (Table 2).

**Table 1:** Prevalence of myopia and high myopia for students from vocational high school and general high school.

	Overall (*n* = 2,867)	Vocational high school (*n* = 814)	General high school (*n* = 2053)	*P* value
Males, no. (%)	1,519 (53.0)	520 (63.9)	999 (48.7)	<0.001^a^
Age, median (IQR), year	16 (2)	17 (1)	16 (2)	<0.001^b^
SE, median (IQR), D	−3.38 (3.75)	−2.50 (3.25)	−3.88 (3.38)	<0.001^b^
Year 10	−3.25 (4.00)	−2.38 (4.19)	−3.63(3.63)	<0.001^b^
Year 11	−3.50 (3.63)	−2.44 (4.38)	−3.88(3.38)	<0.001^b^
Year 12	−3.38 (3.75)	−2.50 (4.38)	−4.00(3.25)	<0.001^b^
Myopia, no. (%)	2,339 (81.6)	571 (70.1)	1768 (86.1)	<0.001^a^
Year 10	747 (80.2)	185 (69.8)	562 (84.3)	<0.001^a^
Year 11	736 (82.1)	153 (72.2)	583 (85.1)	<0.001^a^
Year 12	856 (82.5)	233 (69.1)	623 (88.9)	<0.001^a^
High myopia, no. (%)	337 (11.8)	55 (6.8)	282 (13.7)	<0.001^a^
Year 10	86 (9.2)	16 (6.0)	70 (10.5)	0.034^a^
Year 11	105 (11.7)	15 (7.1)	90 (13.1)	0.016^a^
Year 12	146 (14.1)	24 (7.1)	122 (17.4)	<0.001^a^

^a^*p* value refers to difference between vocational high school and general high school by Chi-square test. ^b^*p* value refers to difference between vocational high school and general high school by Mann–Whitney test.

**Table 2:** Logistic regression results for the students from vocational high school and general high school.

	Myopia			High myopia		
	OR	95%CI	*p* value	OR	95%CI	*p* value
**Overall**						
Age	1.04	0.94–1.14	0.471	1.09	0.97–1.23	0.132
Gender (girls vs. boys)	1.37	1.12–1.67	0.002	1.18	0.94–1.43	0.169
School (GHS vs. VHS)	2.55	2.09–3.11	<0.001	2.19	1.61–3.00	<0.001
**Year 10**						
Age	0.90	0.65–1.26	0.547	0.72	0.40–1.22	0.251
Gender (girls vs. boys)	1.46	1.04–2.05	0.029	1.10	0.70–1.73	0.674
School (GHS vs. VHS)	2.17	1.54–3.05	<0.001	1.65	1.02–2.77	0.048
**Year 11**						
Age	1.07	0.78–1.47	0.673	0.71	0.47–1.06	0.102
Gender (girls vs. boys)	1.63	1.13–2.35	0.009	0.97	0.65–1.50	0.950
School (GHS vs. VHS)	2.03	1.40–2.96	<0.001	1.93	1.08–3.43	0.026
**Year 12**						
Age	0.72	0.52–0.98	0.039	0.65	0.46–0.93	0.018
Gender (girls vs. boys)	1.08	0.78–1.52	0.634	1.31	0.94–1.84	0.111
School (GHS vs. VHS)	3.39	2.42–4.73	<0.001	2.53	1.59–4.02	<0.001

OR, odds ratio; CI, confidence interval; GHS, general high school; VHS, vocational high school.

Besides, the prevalence of myopia and high myopia significant increased with school year in general high school (*p* < 0.05), reaching 84.3% for year 10, 85.1% for year 11, and 87.9% for year 12. The overall prevalence of high myopia, which is 10.5% in year 10, 13.1% in year 11, and 17.4% in year 12, followed the same pattern (Table 3). But there were no significant differences in the prevalence of myopia and high myopia among the three grades in the vocational high school (*p* > 0.05).

**Table 3:** Differences in the prevalence of myopia and high myopia between two educational systems during the school years.

	Overall	*p*-value^a^	Vocational high school	*p*-value^a^	General high school	*p*-value^a^
**Myopia, no. (%)**						
Year 10	747 (80.2)	0.378	185 (69.8)	0.744	562 (84.3)	0.031
Year 11	736 (82.1)		153 (72.2)		583 (85.1)	
Year 12	856 (82.5)		233 (69.1)		623 (88.9)^b^	
**High myopia, no. (%)**						
Year 10	86 (9.2)	0.004	16 (6.0)	0.851	70 (10.5)	0.001
Year 11	105 (11.7)		15 (7.1)		90 (13.1)	
Year 12	146 (14.1)^b^		24 (7.1)		122 (17.4)^b^	

^a^*p* value refers to difference among 3 years by Chi-square test. ^b^Significant to the level of *p* < 0.017, compared with year 10th by Chi-square test (Bonferroni correction).

Distribution of students’ ocular biometric characteristics are shown in Table 3. There were significant differences in the Axial length (AL), Anterior chamber Depth (ACD), Lens Thickness (LT), Lens Position (LP) and Vitreous Chamber Depth (VCD). The general high school students had significantly longer AL (*p* < 0.001), deeper ACD (*p* < 0.001), thinner LT (*p* < 0.001), larger LP (*p* = 0.014) and deeper VCD (*p* < 0.001; Table 4).

**Table 4:** Distribution of students’ ocular biometric characteristics and differences between two educational systems.

	Overall	Vocational high school	General high school	*p-*value
Axial lengths, mean (SD), mm	25.10 (1.24)	24.78 (1.28)	25.22 (1.21)	<0.001
Central corneal thickness, mean (SD), μm	551 (35)	552 (36)	551 (35)	0.576
Anterior chamber depth, mean (SD), mm	3.27 (0.28)	3.24 (0.30)	3.28 (0.27)	<0.001
Lens thickness, mean (SD), mm	3.52 (0.24)	3.55 (0.25)	3.51 (0.23)	<0.001
Lens position, mean (SD), mm	5.03 (0.25)	5.01 (0.26)	5.04 (0.24)	0.014
Vitreous chamber depth, mean (SD), mm	17.75 (1.20)	17.44 (1.22)	17.88 (1.17)	<0.001

*p* value refers to difference in mean between vocational high school and general high school by Student’s *t*-test.

## Discussion

Despite extensive evidence that hereditary factors play a role in the development of myopia (10–13), scientists have seen a major change in the incidence of myopia in various regions of the world throughout the twentieth century, notably in East Asia (3, 14, 15). Intensive near work has long been proposed as a possible mechanism for axial elongation and myopia development (16), with longitudinal studies supporting correlations between near employment and increasing myopia prevalence (17, 18). However, this relationship has been challenged (19, 20). Some research indicated that near-work had a non-significant influence on myopia status (21), myopia incidence (22), or myopia advancement (23). In addition, the direction of causation and the mechanisms involved have been questioned; schooling is a surrogate for near-work activities, but it is difficult to distinguish between reading, writing, and watching electronic screens. (24). Since accommodation occurs during close work in children, ocular changes may establish a reasonable relationship between the development and progression of myopia and close employment (25, 26). Certain near work habits, such as reading continuously for 30 min or more (17, 27, 28) and working at a distance of less than 30 cm (27, 28), have been attributed to myopia in youngsters. Furthermore, Wang et al. (29) discovered that Chinese children had reduced reading distances of 16–28 cm and a larger degree of head tilt when writing than other groups. As a result, the intensity of close work appears to be more significantly linked to myopia prevalence and longer axial length than near work in general.

In addition to behavioral findings, a number of studies have investigated the physiological changes that occur in the eye during close proximity activity. Studies examining modifications in ocular biometry during accommodation over a restricted range of demands have revealed comparable tendencies of decreased anterior chamber depth, vitreous chamber depth, increased lens thickness, and increased axial length in adults. Yet, due to the axial length inaccuracy generated by the increase in crystalline lens thickness, a number of the adult investigations likely overestimated the increase of axial length during accommodation (30). Hughes et al. (31) reported modest axial length increases that occurred during accommodation in toddlers, with a magnitude of change comparable to previous research on adults up to a 6 D demand when lens thickness errors are considered. Moreover, it was discovered that the variations in axial length were closely related to the accommodation-induced changes in lens thickness and anterior chamber depth. Similar patterns were also detected in our data.

In previous studies, myopia appears to be highly associated with educational attainment or intensity (1, 32), better academic achievement (21, 33, 34), and even level of intelligence in prior studies (35, 36). Due to the bidirectional relationship between refractive error and visual acuity on the one hand and academic accomplishment on the other, it is difficult to draw definitive cause-and-effect conclusions from studies (37). For instance, a high prevalence of myopia has been observed in communities that receive intense training at a young age, such as Jewish boys in Israel who underwent Orthodox or traditional Orthodox education (38). In contrast, the prevalence of myopia is mild and equivalent to the majority of western nations among East Asians with less education (39). In recent years, the increasing prevalence of myopia in East and Southeast Asia has prevented the implementation of mass rigorous schooling (40). Mirshahi et al. analyzed the myopia prevalence and severity in relation to years spent in school and level of post-secondary professional education (32). Their findings suggested that more myopic refraction was related to higher degrees of school and post-school professional education. This school-based analysis indicates, to the best of our knowledge, a plausible correlation between myopia prevalence, general secondary school education, and postsecondary professional training. After controlling for age and gender, the association remained strong.

For the first time in our study, we evaluated the effects of two distinct academic expectations and learning styles on the topic of teenage myopia. Initially, we assumed that this was the result of a cumulative effect; however, our investigation proved otherwise. According to the results of three school years in regular high schools, the prevalence of myopia (mild, moderate, and severe) grew dramatically from one school year to the following. However, the prevalence of myopia and high myopia among vocational high school students remained largely consistent as the school year advanced.

Comparing the curricular patterns of the two educational systems reveals substantial differences between general and vocational high schools. To begin with, the general high school teaching model requires pupils to devote the majority of their time to focused close work (except in physical education classes). During a normal day in general high school, Chinese students participate in teaching tasks for an average of 360 min (about two-thirds of the time spent at school) and continuous close tasks for an average of 30 min per task. Recent research on youngsters in Australia and China found that near working distances ranging from 25 cm to less than 10 cm (27, 41). In contrast to traditional high schools, vocational high schools emphasize technological subjects, covering welding technology applications, automotive applications and maintenance, electromechanical technology applications, numerical control technology applications, and so on. This modification could result in less time spent working in close proximity and more time spent engaging in outdoor activities. Secondly, traditional high schools have stricter academic requirements, and students spend more time outside of school completing assignments, whereas vocational high schools are more laid-back. Thirdly, the final year of general high schools typically include more study time preparing for university entrance examination. Comparatively, vocational high schools will have a greater emphasis on internships, and the compulsory academic time will be 4 lectures (approximately 4 h in total) less per school day than that of normal high schools. Therefore, we speculate that, compared to heritable characteristics, recurrent close tasks in schooling may be a more significant factor in the progression of myopia in adolescents aged 15 to 18 years (in school years 10–12).

It is important to note that this research has several limitations. In our investigation, the photoscreener only delivered findings for noncycloplegic refraction. It is beneficial for population-based screening and myopia monitoring due to its high efficiency, but it is not currently regarded as a substitute for cycloplegic refraction. In addition, our study focused on demonstrating the association between myopia and time spent in school among 15 to 18-year-olds, but did not adequately account for time spent after school. With the exception of studying, leisure activities at other times require more investigation. Additional information, including family history, parental myopia, and education level, should be included to the study. The next phase of this study will entail recruiting additional participants and refining the questionnaire in order to compare the behaviors of diverse pupils in greater detail. A longitudinal study of educationally relevant topics will be pursued next.

## Conclusion

Between vocational high school students and general high school students, there are substantial differences in myopia prevalence, spherical equivalent, and other ocular biological parameters. Myopia and high myopia prevalence in general high school students increased significantly with school year, whereas the prevalence among vocational high school students are generally consistent. These findings might provide guidance in the prevention and care of myopia in high school students. Finally, the data reported in this study supplements real-world data from 15- to 18-year-old students, contributing in the standardization of optical modeling of the eyes of this age group.

## Data availability statement

The raw data supporting the conclusions of this article will be made available by the authors, without undue reservation.

## Ethics statement

The studies involving human participants were reviewed and approved by The Medical Ethics Committee of Tianjin Eye Hospital. Written informed consent to participate in this study was provided by the participants’ legal guardian/next of kin.

## Author contributions

YL, DM, RH, and WZ contributed to the conception of the study and performed the data analyses and wrote the manuscript. YL, YW, XW, and CX collected the data. All authors contributed to the article and approved the submitted version.

## Funding

This study was supported by Tianjin Key Research and Development Plan of Science and Technology (19YFZCSY00990), Tianjin Key Medical Discipline (Specialty) Construction Project (TJYXZDXK-016A), Tianjin Eye Hospital Science and Technology Fund Youth Cultivation Project (YKPY2201) and Open Fund of Nankai University Eye Institute Grant (NKYKD202202).

## Conflict of interest

The authors declare that the research was conducted in the absence of any commercial or financial relationships that could be construed as a potential conflict of interest.

## Publisher’s note

All claims expressed in this article are solely those of the authors and do not necessarily represent those of their affiliated organizations, or those of the publisher, the editors and the reviewers. Any product that may be evaluated in this article, or claim that may be made by its manufacturer, is not guaranteed or endorsed by the publisher.

## References

[1] Morgan IG Ohno-Matsui K Saw SM. Myopia. Lancet. (2012) 379:1739–48. doi: 10.1016/S0140-6736(12)60272-4, PMID: 22559900

[2] Holden BA Fricke TR Wilson DA Jong M Naidoo KS Sankaridurg P. Global prevalence of myopia and high myopia and temporal trends from 2000 through 2050. Ophthalmology. (2016) 123:1036–42. doi: 10.1016/j.ophtha.2016.01.006, PMID: 26875007

[3] Morgan I Rose K. How genetic is school myopia? Prog Retin Eye Res. (2005) 24:1–38. doi: 10.1016/j.preteyeres.2004.06.004, PMID: 15555525

[4] Saw SM Hong CY Chia KS Stone RA Tan D. Near work and myopia in young children. Lancet. (2001) 357:390. doi: 10.1016/S0140-6736(05)71520-8, PMID: 11211020

[5] Wang J Ying GS Fu X Zhang R Meng J Gu F. Prevalence of myopia and vision impairment in school students in eastern China. BMC Ophthalmol. (2020) 20:2. doi: 10.1186/s12886-019-1281-0, PMID: 31898504 PMC6941318

[6] Xiong S Zhang B Hong Y He X Zhu J Zou H. The associations of lens power with age and axial length in healthy Chinese children and adolescents aged 6 to 18 years. Invest Ophthalmol Vis Sci. (2017) 58:5849–55. doi: 10.1167/iovs.17-22639, PMID: 29141080

[7] Naroo S. An increase in interest in myopia control. Cont Lens Anterior Eye. (2020) 43:1–2. doi: 10.1016/j.clae.2019.12.010, PMID: 31859146

[8] Qian X Li Y Ding G Li J Lv H Hua N. Compared performance of spot and SW800 photoscreeners on Chinese children. Br J Ophthalmol. (2019) 103:517–22. doi: 10.1136/bjophthalmol-2018-311885, PMID: 29986946

[9] Flitcroft DI He M Jonas JB Jong M Naidoo K Ohno-Matsui K. IMI - defining and classifying myopia: a proposed set of standards for clinical and epidemiologic studies. Invest Ophthalmol Vis Sci. (2019) 60:M20–30. doi: 10.1167/iovs.18-25957, PMID: 30817826 PMC6735818

[10] Kiefer AK Tung JY Do CB Hinds DA Mountain JL Francke U. Genome-wide analysis points to roles for extracellular matrix remodeling, the visual cycle, and neuronal development in myopia. PLoS Genet. (2013) 9:e1003299. doi: 10.1371/journal.pgen.1003299, PMID: 23468642 PMC3585144

[11] Verhoeven VJ Hysi PG Wojciechowski R Fan Q Guggenheim JA Hohn R. Genome-wide meta-analyses of multiancestry cohorts identify multiple new susceptibility loci for refractive error and myopia. Nat Genet. (2013) 45:314–8. doi: 10.1038/ng.2554, PMID: 23396134 PMC3740568

[12] Verhoeven VJ Hysi PG Saw SM Vitart V Mirshahi A Guggenheim JA. Large scale international replication and meta-analysis study confirms association of the 15q14 locus with myopia. CREAM Consortium Hum Genet. (2012) 131:1467–80. doi: 10.1007/s00439-012-1176-0, PMID: 22665138 PMC3418496

[13] Young TL. Molecular genetics of human myopia: an update. Optom Vis Sci. (2009) 86:E8–E22. doi: 10.1097/OPX.0b013e3181940655, PMID: 19104467 PMC3718050

[14] Parssinen O. The increased prevalence of myopia in Finland. Acta Ophthalmol. (2012) 90:497–502. doi: 10.1111/j.1755-3768.2011.02210.x, PMID: 21902818

[15] Vitale S Sperduto RD Ferris FL 3rd. Prevalence of refractive error in the united between 1971-1972 and 1999-2004. Arch Ophthalmol. (2009) 127:1632–9. doi: 10.1001/archophthalmol.2009.303, PMID: 20008719

[16] Goss DA. Near work and myopia. Lancet. (2000) 356:1456–7. doi: 10.1016/S0140-6736(00)02864-6, PMID: 11081523

[17] You X Wang L Tan H He X Qu X Shi H. Near work related behaviors associated with myopic shifts among primary school students in the Jiading District of Shanghai: a school-based one-year cohort study. PLoS One. (2016) 11:e0154671. doi: 10.1371/journal.pone.0154671, PMID: 27139017 PMC4854402

[18] Huang HM Chang DS Wu PC. The association between near work activities and myopia in children-a systematic review and meta-analysis. PLoS One. (2015) 10:e0140419. doi: 10.1371/journal.pone.0140419, PMID: 26485393 PMC4618477

[19] Jones-Jordan LA Sinnott LT Graham ND Cotter SA Kleinstein RN Manny RE. The contributions of near work and outdoor activity to the correlation between siblings in the collaborative longitudinal evaluation of ethnicity and refractive error (CLEERE) study. Invest Ophthalmol Vis Sci. (2014) 55:6333–9. doi: 10.1167/iovs.14-14640, PMID: 25205866 PMC4193758

[20] Lin Z Vasudevan B Jhanji V Mao GY Gao TY Wang FH. Near work, outdoor activity, and their association with refractive error. Optom Vis Sci. (2014) 91:376–82. doi: 10.1097/OPX.0000000000000219, PMID: 24637483

[21] Mutti DO Mitchell GL Moeschberger ML Jones LA Zadnik K. Parental myopia, near work, school achievement, and children's refractive error. Invest Ophthalmol Vis Sci. (2002) 43:3633–40. 12454029

[22] Jones-Jordan LA Mitchell GL Cotter SA Kleinstein RN Manny RE Mutti DO. Visual activity before and after the onset of juvenile myopia. Invest Ophthalmol Vis Sci. (2011) 52:1841–50. doi: 10.1167/iovs.09-4997, PMID: 20926821 PMC3101696

[23] Saw SM Nieto FJ Katz J Schein OD Levy B Chew SJ. Factors related to the progression of myopia in Singaporean children. Optom Vis Sci. (2000) 77:549–54. doi: 10.1097/00006324-200010000-00009, PMID: 11100893

[24] Mutti DO Zadnik K. Has near work's star fallen? Optom Vis Sci. (2009) 86:76–8. doi: 10.1097/OPX.0b013e31819974ae, PMID: 19156003

[25] Charman WN. Near vision, lags of accommodation and myopia. Ophthalmic Physiol Opt. (1999) 19:126–33. doi: 10.1046/j.1475-1313.1999.00414.x, PMID: 10615448

[26] Gwiazda J Bauer J Thorn F Held R. A dynamic relationship between myopia and blur-driven accommodation in school-aged children. Vis Res. (1995) 35:1299–304. doi: 10.1016/0042-6989(94)00238-h, PMID: 7610590

[27] Li SM Li SY Kang MT Zhou Y Liu LR Li H. Anyang childhood eye study G. near work related parameters and myopia in Chinese children: the Anyang childhood eye study. PLoS One. (2015) 10:e0134514. doi: 10.1371/journal.pone.0134514, PMID: 26244865 PMC4526691

[28] Ip JM Saw SM Rose KA Morgan IG Kifley A Wang JJ. Role of near work in myopia: findings in a sample of Australian school children. Invest Ophthalmol Vis Sci. (2008) 49:2903–10. doi: 10.1167/iovs.07-0804, PMID: 18579757

[29] Wang Y Bao J Ou L Thorn F Lu F. Reading behavior of emmetropic schoolchildren in China. Vis Res. (2013) 86:43–51. doi: 10.1016/j.visres.2013.03.007, PMID: 23602999

[30] Atchison DA Smith G. Possible errors in determining axial length changes during accommodation with the IOL master. Optom Vis Sci. (2004) 81:283–6. doi: 10.1097/00006324-200404000-00015, PMID: 15097771

[31] Hughes RPJ Read SA Collins MJ Vincent SJ. Changes in ocular biometry during short-term accommodation in children. Ophthalmic Physiol Opt. (2020) 40:584–94. doi: 10.1111/opo.12711, PMID: 32654281

[32] Mirshahi A Ponto KA Hoehn R Zwiener I Zeller T Lackner K. Myopia and level of education: results from the Gutenberg health study. Ophthalmology. (2014) 121:2047–52. doi: 10.1016/j.ophtha.2014.04.017, PMID: 24947658

[33] Saw SM Cheng A Fong A Gazzard G Tan DT Morgan I. School grades and myopia. Ophthalmic Physiol Opt. (2007) 27:126–9. doi: 10.1111/j.1475-1313.2006.00455.x, PMID: 17324201

[34] Morgan IG Rose KA. Myopia and international educational performance. Ophthalmic Physiol Opt. (2013) 33:329–38. doi: 10.1111/opo.12040, PMID: 23662964

[35] Saw SM Tan SB Fung D Chia KS Koh D Tan DT. IQ and the association with myopia in children. Invest Ophthalmol Vis Sci. (2004) 45:2943–8. doi: 10.1167/iovs.03-1296, PMID: 15326105

[36] Teasdale TW Fuchs J Goldschmidt E. Degree of myopia in relation to intelligence and educational level. Lancet. (1988) 2:1351–4. doi: 10.1016/s0140-6736(88)90880-x, PMID: 2904062

[37] Simons HD. An analysis of the role of vision anomalies in reading interference. Optom Vis Sci. (1993) 70:369–73. doi: 10.1097/00006324-199305000-00005, PMID: 8515964

[38] Bez D Megreli J Bez M Avramovich E Barak A Levine H. Association between type of educational system and prevalence and severity of myopia among male adolescents in Israel. JAMA Ophthalmol. (2019) 137:887–93. doi: 10.1001/jamaophthalmol.2019.1415, PMID: 31145422 PMC6547130

[39] Vitale S Ellwein L Cotch MF Ferris FL 3rd Sperduto R. Prevalence of refractive error in the United States, 1999-2004. Arch Ophthalmol. (2008) 126:1111–9. doi: 10.1001/archopht.126.8.1111, PMID: 18695106 PMC2772054

[40] Morgan IG French AN Ashby RS Guo X Ding X He M. The epidemics of myopia: aetiology and prevention. Prog Retin Eywe Res. (2018) 62:134–49. doi: 10.1016/j.preteyeres.2017.09.004, PMID: 28951126

[41] Narayanasamy S Vincent SJ Sampson GP Wood JM. Visual demands in modern Australian primary school classrooms. Clin Exp Optom. (2016) 99:233–40. doi: 10.1111/cxo.12365, PMID: 26889920

